# Job insecurity during the COVID-19 pandemic and counterproductive work behavior: The sequential mediation effects of job stress and organizational identification and the buffering role of corporate social responsibility

**DOI:** 10.3389/fpubh.2022.1037184

**Published:** 2023-02-17

**Authors:** Byung-Jik Kim, Julak Lee, Jeyong Jung, Min-Jik Kim

**Affiliations:** ^1^College of Business, University of Ulsan, Ulsan, South Korea; ^2^Department Psychology, Yonsei University, Seoul, South Korea; ^3^Department of Industrial Security, Chung-Ang University, Seoul, South Korea; ^4^Department of Police Science, University of Ulsan, Ulsan, South Korea; ^5^School of Industrial Management, Korea University of Technology and Education, Cheonan, South Korea

**Keywords:** job insecurity, counterproductive work behavior, job stress, organizational identification, CSR activities, moderated sequential mediation model

## Abstract

Swift social and economic environmental changes such as those associated with the COVID-19 pandemic have led to decreased job security. Although numerous previous studies have examined the influence of job insecurity on employee perceptions, attitudes, and behaviors, the link between job insecurity and negative behavior and its underlying or intermediating mechanisms remain underexplored. The significance of an organization's positive behaviors, which fall under the umbrella of corporate social responsibility (CSR), also deserves more attention. To address these gaps, we examined both the mediator and the moderator in the association between job insecurity and negative employee behavior by establishing a moderated sequential mediation model. We hypothesized that the levels of employee job stress and organizational identification sequentially mediate the relationship between job insecurity and counterproductive work behavior as a representative negative behavior. We also hypothesized that CSR activities play a buffering role that moderates the influence of job insecurity on job stress. We used three-wave time-lagged data collected from 348 employees in South Korean organizations to demonstrate that job stress and organizational identification sequentially mediate the relationship between job insecurity and counterproductive work behavior, and that CSR activities function as a buffering factor that decreases the influence of job insecurity on job stress. The results of this research suggest that the levels of job stress and organizational identification (as sequential mediators) as well as CSR activities (as a moderator) are underlying mechanisms in the link between job insecurity and counterproductive work behavior.

## Introduction

Swift social and economic environmental changes brought about by events including the COVID-19 pandemic, the artificial intelligence (AI) revolution, and robot processing automation (RPA) can constitute great shocks, causing recession, and economic crisis. In order to effectively respond to such unexpected changes, organizations tend to implement massive restructuring and downsizing, causing their employees to experience high levels of job insecurity (1, 2). Job insecurity is defined as an employee's perception or belief about the uncertainty his or her employment (3). Previous studies reported that job insecurity crucially affects a variety of organizational outcomes by playing the role of a severe job stressor. For example, job insecurity has been known to substantially predict poor employee mental/physical health, perceptions, attitudes, and behaviors (e.g., job satisfaction, perceived organizational support, organizational commitment, organizational identification, organizational trust, employee engagement, creativity, and organizational citizenship behavior), and poor organizational-level outcomes (3–10). Although many studies of job insecurity have delved into the impacts of job insecurity on critical organizational outcomes, important research gaps remain (6, 9).

First, extant studies of the relationships between job insecurity and organizational outcomes are inconclusive (5, 6, 9, 11). For instance, meta-analyses showed that an unstable job substantially decreases the quality of individual-level outcomes (5). The harmful effects originate in the finding that job instability is likely to play a role-boosting factor that drastically increases employee stress and negative emotions (4, 8, 9, 11). In contrast, other studies have revealed that job instability tends to enhance the quality of employee outcomes or performance in an organization. This interesting phenomenon is based on the efforts of employees in response to job insecurity to preserve their job in the organization (10). Furthermore, research on job insecurity has shown that an unstable job is not related to employee outcomes (10, 12). These inconclusive results originate in the lack of studies on the intermediating or underlying mechanisms (i.e., mediators and moderators) of this link (9). Thus, work on the intermediating processes is critical.

Second, previous studies paid relatively less attention to employees' “negative behaviors” such as deviant or counterproductive work behavior (6, 9, 11). The extant research has mainly focused on employees' “positive” perceptions, attitudes, and behaviors such as job satisfaction, organizational commitment, organizational identification, organizational trust, voice/safety behavior, and organizational citizenship behavior (3–11). We acknowledge that positive perceptions, attitudes, and behaviors are crucial factors to determine organizational survival by significantly affecting organizational performance. However, considering that organizational life includes both positive and negative sides and that positive and negative aspects pertinent to an employee's behaviors tend to possess different psychological mechanisms, understanding the influence of job insecurity on negative behaviors is important (6, 9, 13).

Third, extant studies of job insecurity have ignored the significance of organizational positive and benevolent behaviors toward society such as corporate social responsibility (CSR) activities (6, 9, 11). Although some studies have revealed a variety of contextual variables that moderates the influences of an unstable job on organizational outcomes at the macro-economic level (e.g., labor market condition, social safety network), organizational-level (e.g., previous financial performance, productivity, and quality of organizational communication), and individual-level (e.g., employee self-efficacy, proactive coping, and job involvement), those studies did not focus on the organization's “goodness,” which is one of the most essential values in human society (6, 9). Considering that kindness and goodness (e.g., benevolent activities for society) are likely to have healing effects for human beings, it is important to investigate their moderating role.

To deal with these research gaps, we investigate the mechanisms intermediating between employee job insecurity and counterproductive work behavior (CWB) as a negative behavior in an organization. CWB can be defined as intentional action by an employee that directly/indirectly harms coworkers, customers, and the organization itself (14, 15). Employee job insecurity is a critical antecedent of CWB (16, 17). An employee who feels a sense of job insecurity tends to experience serious job stress (18, 19). Such stress may motivate employees to take revenge upon the organization by engaging in CWB (15).

In specific, we suggest that employee job stress and organizational identification sequentially mediate the association between job insecurity and CWB. Moreover, corporate social responsibility (CSR) may play a buffering role in the job insecurity-job stress link by moderating this relationship.

Job insecurity would increase employee job stress. Job stress refers to negative psychological states or negative responses toward various stimuli (i.e., job stressors) such as anxiety, anger, and depression (20). Extant research demonstrated that employee job insecurity functions as one of the most serious and critical job stressors and drastically increases levels of stress at work (6, 9, 11, 18). Based on social exchange theory (21), we suggest that employee job stress is likely to decrease organizational identification (22–24). Such identification is the degree to which an employee considers himself or herself to be one with his or her organization, functioning as a “root construct” in an organization to facilitate the quality of critical organizational outcomes (23, 24).

We propose that an employee's organizational identification is negatively associated with the level of his or her CWBs. According to social identity theory (23–25), an employee with a low level of organizational identification due to high job stress is not likely to believe that the success of his or her organization is directly related to his or her own self-concept. Thus, the employee may not facilitate behaviors to contribute to the achievement of the organization's success or to stop engaging in behaviors that detract from this goal. Eventually, he or she fails to reduce actions harmful to the organization such as CWB or even increases such negative behaviors (26–28).

We use the context–attitude–behavior perspective to integrate the relationships among job insecurity, job stress, organizational identification, and counterproductive work behavior (29) by applying it to our sequential mediation model. This perspective proposes that a variety of social and contextual factors such as rules, systems, cultures, and climates exist in an organization and plays critical roles in building employee attitudes, eventually influencing employee behaviors. Job insecurity is a crucial social context that affects employee attitudes such as job stress and organizational identification. These attitudes are likely to lead to behavior such as CWB. Relying on these arguments, in this research we propose that employee job stress and organizational identification sequentially mediate the relationship between job insecurity and CWB.

Furthermore, we suggest that CSR functions as an important moderating factor that buffers the negative influence of job insecurity on job stress. As mentioned above, our argument that employee job insecurity increases job stress may be reasonable, but the influence of job insecurity on job stress may not apply to all situations or contexts in the same way since a variety of contextual/moderating factors directly/indirectly affects the job insecurity-job stress link in real organizations. Among many potential moderators, we propose that CSR, an organizational-level benevolent activity, plays a critical moderating role by buffering the negative impact of job insecurity on job stress. CSR describes an organization's moral efforts to facilitate the welfare of many stakeholders, including shareholders, employees, customers, suppliers, the government, and the environment itself (13, 30–33).

For example, when a firm proactively implements CSR, even if an employee of the firm feels a sense of job insecurity, the negative influence of the unstable job is likely to be decreased because the employee is more likely to perceive that his or her organization is respectable or reputable. Then, the employee may feel a sense of escalated social self (23–25), eventually enhancing their pride, identification, and commitment toward his or her organization (13, 30). These positive psychological states may function as a buffering factor by reducing the negative impacts of job insecurity. In other words, the positive psychological states that originate in CSR are likely to offset the negative influence of an unstable job. In contrast, when a firm passively or rarely implements CSR, an employee who is suffering from negative feelings such as anxiety, fear, or stress from job insecurity may not have an opportunity to enhance his or her social self, pride, and positive perceptions toward his or her organization. As a result, the negative influence of job insecurity may not be resolved and could even be amplified (13, 26, 30, 33)].

**Figure 1:**
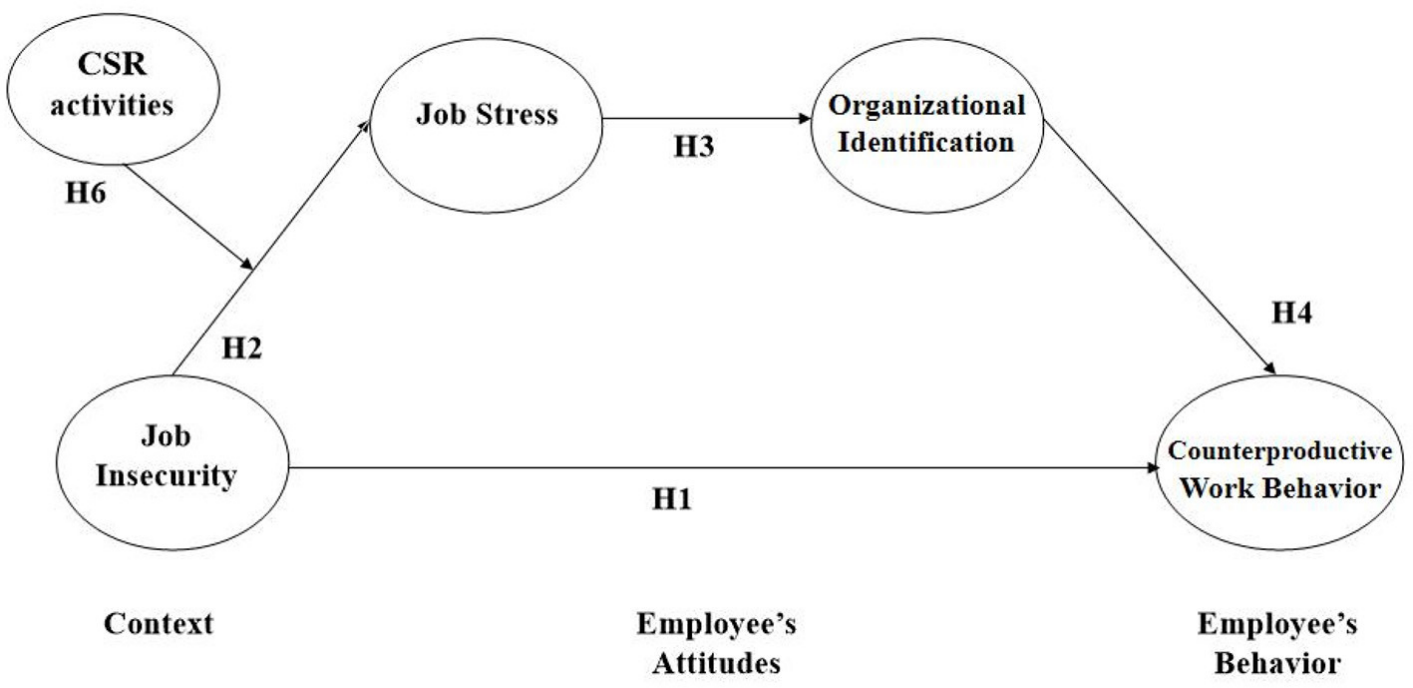
Theoretical model.

Based on those findings, we attempt to analyze the influence of job insecurity on CWB through the sequential mediating effects of job stress and organizational identification. Moreover, we also propose that CSR practices play a contingent role that moderates the association between job insecurity and job stress (please see Figure 1). To empirically test our hypotheses, we built a moderated sequential mediation model with structural equation modeling (SEM) using three wave time lagged data from 348 Korean workers. We expect that this research will contribute to the literature on job insecurity and CWB as follows. First, we elucidated the inconclusive relationship between job insecurity and organizational outcomes by investigating intermediating mechanisms (i.e., mediators and moderators). Second, we found that CSR activities, as organizational-level positive and benevolent actions, play a contextual role that moderates the job insecurity-job stress link. Third, we delved into the influence of job insecurity on negative employee behaviors such as counterproductive work behavior, rather than positive perceptions or attitudes. Last, from a methodological perspective, we tried to complement the limitations of cross-sectional data by applying a longitudinal approach (i.e., 3-wave time-lagged research design).

***Hypothesis 1:*** An employee's job insecurity may increase his or her CWB.

***Hypothesis 2:*** An employee's job insecurity may increase his or her job stress.

***Hypothesis 3:*** An employee's job stress may decrease his or her organizational identification.

***Hypothesis 4:*** An employee's organizational identification may decrease his or her CWB.

***Hypothesis 5:*** Employee's job stress and organizational identification sequentially mediate the relationship between job insecurity and CWB.

***Hypothesis 6:*** CSR moderates the relationship between job insecurity and job stress.

## Methods

### Participants and procedures

We surveyed current employees of organizations in South Korea over 19 years of age across three time points. They were recruited through an online survey company offering the largest population of research panelists in Korea, ~3,460,000 potential respondents. The participants reported their occupation status when they registered for online membership *via* a user authentication system (e.g., cellular phone number or email address). Such online survey systems have been shown to be reliable (34).

By collecting data for three time periods, we address the fundamental issue embedded in cross-sectional research design. The online system allowed us to track who responded to our survey, confirming lack of difference in participants from time point 1 to time point 3. The interval between surveys was 4 or 5 weeks. Our survey system was open for 2 or 3 days each at each time point to provide enough time for participants to respond. When the system was open, participants could access it at any time. The company monitored the integrity of data using traps for geo-IP violators and timestamps to flag efficient response. These safeguards restricted participants from logging into the survey site and filling out multiple surveys.

Experts in the research firm directly contacted participants to ask for permission to include them in our survey, assuring them that their participation was voluntary and their responses would remain confidential and only be used for research purposes. The company obtained both informed consent and agreement for compliance with ethical requirements from participants. The company provided the participants with rewards for their participation in the form of cash (US $8). This research was approved by the institutional review board (IRB) of one of the participating universities in South Korea.

The research company randomly chose participants through stratified sampling to decrease the possibility of sampling bias. Stratified random sampling reduces bias due to employee characteristics that may influence the results of research (e.g., gender, age, position, education, and industry type). Using online survey tools, we were able to verify the lack of difference in respondents from time point one to time point three.

At time point 1, 512 employees participated in our survey; at time point 2, 421 workers participated in the second survey; and at time point 3, 357 employees responded to the third survey. To determine the sample size, we consulted previous research. First, we calculated the minimum sample size using G^*^Power version 3.1.9.7. A power analysis using this program demonstrated that a sample size of 348 provided sufficient power (≥0.80) to detect a medium effect with an alpha level of *p* = 0.05 (35). In addition, Barclay et al. (36) suggested that one observable variable must be analyzed in at least 10 cases (i.e., the rule of 10) with SEM. Because this study includes 22 observable variables, our 348 cases (response rate: 67.97%) comprise an adequate sample. The time intervals among the time points were ~5 or 6 weeks. We deleted missing data from the raw material after 6 weeks. The characteristics of the sample are described in Table 1.

**Table 1:** Descriptive characteristics of the study sample.

**Characteristic**	**Percent**
**Gender**
Male	50.0%
Female	50.0%
**Age (years)**
20–29	14.7%
30–39	35.3%
40–49	33.9%
50–59	16.1%
**Education**
Less than high school	8.6%
Community college	19.3%
Bachelor's degree	59.8%
Master's degree or higher	12.4%
**Occupation**
Office worker	71.3%
Professional (Practitioner)	7.2%
Public official	6.0%
Manufacturing	5.7%
Sales and marketing	4.3%
Administrative	4.0%
Education	0.3%
Others	1.2%
**Position**
Staff	25.0%
Assistant manager	21.6%
Manager or deputy general manager	31.9%
Department/general manager or director and above	21.5%
**Tenure (years)**
< 5	46.8%
5–10	27.1%
11–15	12.9%
16–20	7.5%
21–25	2.3%
More than 26	2.9%
**Industry type**
Manufacturing	24.4%
Services	18.6%
Construction	11.9%
Health and welfare	10.2%
Information services and telecommunications	8.7%
Education	8.4%
Financial/insurance	4.1%
Consulting and advertising	1.2%
Others	12.5%

### Measures

The survey measured distinct variables in our research model at each time point. At time point 1, the respondents were asked about job insecurity and CSR activities. At time point 2, we measured degrees of job stress and organizational identification. At time point 3, we collected data about participants' CWB. These variables were assessed through multi-item scales on a five-point Likert scale (1 = strongly disagree, 5 = strongly agree). We computed the internal consistency of each variable using Cronbach's alpha.

### Job insecurity (time point 1, collected from employees)

We used four items for the job insecurity scale (37). Sample items were “If my current organization were facing economic problems, my job would be the first to go,” “I will not be able to keep my present job as long as I wish,” and “My job is not a secure one.” The Cronbach's alpha value was 0.90.

### CSR (time point 1, collected from employees)

The CSR was measured using 12 items from the CSR scale suggested by Farooq et al. (38), which originated in Turker's CSR measure (39). The scale we utilized in this study consists of four dimensions for measuring CSR activities: (1) environment, (2) community, (3) employee, and (4) customer. Each of the four domains includes three items and indicates the corresponding stakeholders in the organization's social responsibility. For the environment domain, a sample item is “our organization participates in activities that aim to protect and improve the quality of the natural environment.” For the community domain, a sample item is “our organization contributes to campaigns and projects that promote the wellbeing of society.” For the employee domain, a sample item is “our organization supports employee growth and development.” For the customer domain, a sample item is “our organization respects consumer rights beyond legal requirements.” All items were previously used in empirical studies conducted in South Korean contexts [e.g., (7, 40)]. The Cronbach's alpha value was 0.91.

### Job stress (time point 2, collected from employees)

To measure levels of employee job stress, we used four items of a job stress scale adapted from previous work (7, 41). Sample items were “At work, I felt stressed during the last 30 days,” “At work, I felt anxious during the last 30 days,” and “At work, I felt frustrated during the last 30 days.” The Cronbach's alpha value was 0.89.

### Organizational identification (time point 2, collected from employees)

To measure the degree of employee organizational identification, we utilized five items from Mael and Ashforth (42). Some sample items are “When someone criticizes my organization, it feels like a personal insult” and “My organization's successes are my successes.” The Cronbach's alpha value was 0.84.

### Counterproductive work behavior (time point 3, collected from employee supervisors)

The degree of CWB was measured through five items of the CWB scale by Fox et al. (14). The employees' immediate supervisor evaluated the level of employee CWB. A sample is “This employee purposely worked slowly when things needed to get done.” The Cronbach's alpha value was 0.91.

### Control variables

Based on previous research (43), the dependent variable, CWB, was impacted by factors such as tenure, gender, position, and education of an employee. The control variables were collected at time point 2.

### Statistical analysis

First, frequency analysis was performed to assess the participants' demographic features. We conducted Pearson correlation analysis in SPSS 26 to compute the relationships among our research variables. Then, following Anderson and Gerbing (44), we used a two-step approach that consists of (1) measurement and (2) application of the structural model. To test the validity of the measurement model, we performed confirmatory factor analysis (CFA). Next, based on SEM, a moderated mediation model with the maximum likelihood (ML) estimator was used with the AMOS 23 program to test the structural model.

To test whether various model fit indexes were acceptable, we utilized a variety of goodness-of-fit indices including the comparative fit index (CFI), the Tucker–Lewis index (TLI), and the root mean square error of approximation (RMSEA). Previous research reported that CFI and TLI values >0.90 and RMSEA values < 0.06 are appropriate (45). A bootstrapping analysis was implemented to test whether the indirect effect was significant (46) and whether our mediation hypothesis was supported with a 95% bias-corrected confidence interval (CI). If the CI does not include zero (0), this indicates that the indirect effect is statistically significant at the 0.05 level (46).

## Results

### Descriptive statistics

Our research variables, including job insecurity, CSR, job stress organizational identification, and CWB, were significantly related. The correlation analysis results are shown in Table 2.

**Table 2:** Correlations among research variables.

	**Mean**	**S.D**.	**1**	**2**	**3**	**4**	**5**	**6**	**7**	**8**
1. Gender_T2	1.50	0.50	–							
2. Education_T2	2.76	0.78	−0.14**	–						
3. Tenure_T2	7.42	7.18	−0.25**	0.02	–					
4. Position_T2	2.90	1.61	−0.43**	0.21**	0.28**	–				
5. Job insecurity_T1	2.78	0.82	−0.11**	−0.06	−0.003	0.11*	–			
6. CSR_T1	3.19	0.68	−0.17**	0.13*	0.18**	0.16**	−0.13*	–		
7. Job Stress_T2	2.93	0.76	−0.001	−0.09	0.22	−0.06	0.25**	−0.11*	–	
8. OI_T2	3.42	0.71	−0.17**	0.92	0.14*	0.20**	−0.08	0.41**	−0.18**	–
9. CWB_T3	2.31	0.77	−0.10	−0.93	0.70	−0.07	0.18**	−0.08	0.26**	−0.19**

*p < 0.05. **p < 0.01. S.D, deviation; CSR, corporate social responsibility; OI, organizational identification; CWB, counterproductive work behavior.

### Measurement model

To test the discriminant validity of the main research variables (job insecurity, CSR, job stress, organizational identification, and CWB), we performed a CFA for all items by assessing the measurement model's goodness-of-fit. To be specific, we compared our hypothesized model, a 5-factor model (job insecurity, CSR, job stress, organizational identification, and CWB), to other alternative models with fewer factors using a series of chi-square difference tests.

First, the hypothesized 5-factor model had a good and acceptable fit [χ^2^ (df = 94) = 186.013; CFI = 0.970; TLI = 0.962; RMSEA = 0.053]. Then, we conducted a series of chi-square difference tests by comparing the 5-factor model to a 4-factor model [χ^2^ (df = 98) = 732.341; CFI = 0.795; TLI = 0.749; RMSEA = 0.137], a 3-factor model [χ^2^ (df = 101) = 1,326.855; CFI = 0.604; TLI = 0.529; RMSEA = 0.187], a 2-factor model [χ^2^ (df = 103) = 1,811.057; CFI = 0.448; TLI = 0.356; RMSEA = 0.219], and a 1-factor model [χ^2^ (df = 104) = 2,372.264; CFI = 0.266; TLI = 0.154; RMSEA = 0.251]. The 5-factor model was better than all others, indicating that the five variables have an appropriate degree of discriminant validity.

### Structural model

The current study includes a moderated sequential mediation model of the job insecurity-CWB link. In the sequential mediation structure, the job insecurity-CWB link is sequentially mediated by degree of employee job stress and organizational identification. In the moderation structure, CSR activities function as a buffering factor that moderates the impact of job insecurity on job stress.

Next, in the moderation structure, we multiplied the two variables (i.e., job insecurity and CSR) to form an interaction term. Before multiplication, the two variables were centered on their respective means to increase the validity of the moderation analysis by diminishing the degree of multi-collinearity between variables and minimizing the loss of correlations (47).

To test the impact of multicollinearity bias, we measured the values of variance inflation factors (VIF) and tolerances (47). The VIF values for job insecurity and CSR were 1.02 and 1.02, respectively. The values of tolerance were 0.98 and 0.98, respectively. The finding of VIF values smaller than 10 with tolerance values above 0.2 indicates that job insecurity and CSR are relatively free from the multi-collinearity issue.

### Results of mediation analysis

To determine the best mediation model, we compared a full mediation model to a partial mediation model using a chi-square difference test. The full mediation model is identical to the partial mediation model except that it includes a direct path from job insecurity to counterproductive work behavior. The fit indices of both the full mediation model [χ^2^ = 259.476 (df = 118), CFI = 0.949, TLI = 0.933, and RMSEA = 0.059] and the partial mediation model [χ^2^ = 251.185 (df = 117), CFI = 0.951, TLI = 0.936, and RMSEA = 0.057] were acceptable. However, the chi-square difference test between the models [Δ$\chi_{\left(1\right)}^{2}$ = 8.291, *p* < 0.01] demonstrated that the partial mediation model was superior and indicates that job insecurity influences CWB indirectly rather than directly.

**Figure 2:**
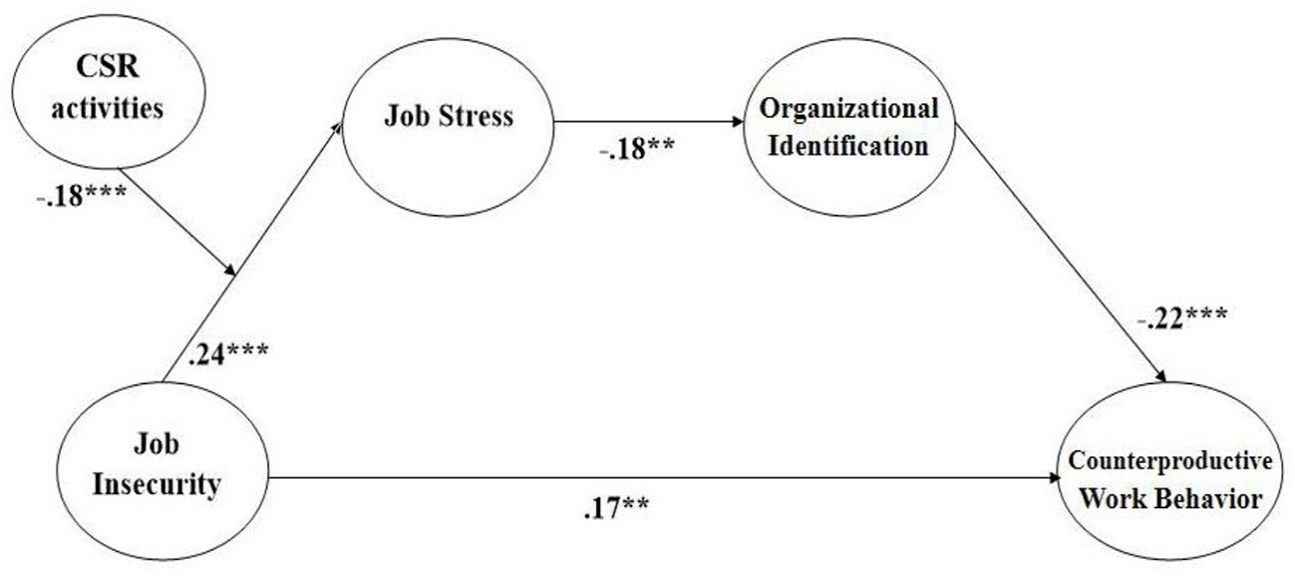
Coefficient values of our research model (**p < 0.01, ***p < 0.001. All values are standardized).

Control variables, such as tenure, education, and position, were included in the research model of the dependent variable, CWB. Only position (β = −0.12, *p* < 0.05) and gender (β = −0.12, *p* < 0.05) were statistically significant. By including the control variables, our research model showed that job insecurity is significantly and positively associated with CWB (β = 0.17, *p* < 0.01), supporting Hypothesis 1; that job insecurity is significantly and positively associated with job stress (β = 0.24, *p* < 0.001), supporting Hypothesis 2; that job stress is significantly and negatively associated with organizational identification (β = −0.18, *p* < 0.01), supporting Hypothesis 3; and that organizational identification is significantly and negatively related to CWB (β = −0.22, *p* < 0.001), supporting Hypothesis 4 (Table 3, Figure 2).

**Table 3:** Results of the structural model.

**Hypothesis**	**Path (Relationship)**	**Estimate**	**S.E**.	**Standardized estimate**	**Supported**
1	Job insecurity → CWB	0.142	0.049	0.167**	Yes
2	Job insecurity → Job stress	0.208	0.049	0.241***	Yes
3	Job stress → OI	−0.154	0.052	−0.178**	Yes
4	OI → CWB	−0.251	0.067	−0.220***	Yes
6	Job insecurity × CSR	−0.209	0.063	−0.181***	Yes

**p < 0.01. ***p < 0.05. Estimate indicates standardized coefficients. SE, standard error.

### Bootstrapping

To test the sequential mediation effects of job stress and organizational identification in the job insecurity-CWB link (Hypothesis 4), we conducted a bootstrapping analysis with a sample of 10,000 (46). The indirect mediation effect would be significant at a 5% level if the 95% bias-corrected confidence interval (CI) for the effect of mean indirect mediation excludes 0 (46).

The bias-corrected CI for the mean indirect effect did not include 0 [95% CI = (0.002, 0.025)]. This means that that the indirect sequential mediation effects of job stress and organizational identification were statistically significant, supporting Hypothesis 5. The direct, indirect, and total effects of the paths from job insecurity to CWB are shown in Table 4.

**Table 4:** Direct, indirect, and total effects of the final research model.

**Model (Hypothesis 5)**	**Direct effect**	**Indirect effect**	**Total effect**
Job insecurity → Job stress → OI → CWB	0.166	0.100	0.176

All values are standardized.

### Moderation analysis

**Figure 3:**
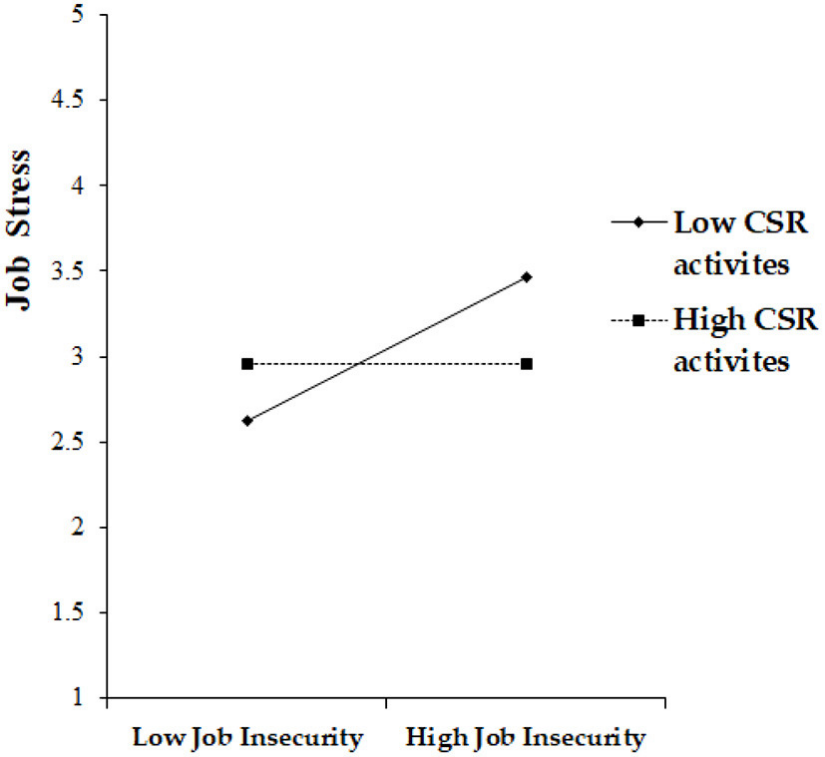
Moderating effect of CSR in the job insecurity–Job stress link.

We tested the moderation effect of CSR activities on the relationship between job insecurity and job stress through a mean-centering process using an interaction term. The coefficient of the interaction term (β = −0.18, *p* < 0.001) was statistically significant. This means that CSR activities moderate the relationship between job insecurity and job stress by playing a buffering role. When the level of CSR is high, the impact of job insecurity on job stress is decreased, supporting Hypothesis 6 (please see Figure 3).

## Discussion

Using 3-wave time-lagged data obtained for 348 employees in South Korea, we demonstrated that employee job stress and organizational identification function as sequential mediators in the job insecurity-CWB link. Moreover, we determined that CSR plays a buffering role that reduces the negative impact of job insecurity on job stress. In the following sections, we describe the theoretical/practical implications and limitations of this paper and suggest ideas for future research.

### Theoretical implications

We expect that the current research can contribute to the job insecurity literature from the theoretical point of view. First, to address the issue of inclusive results between job insecurity and organizational outcomes, we investigated the intermediating processes (i.e., mediators and moderators) (9). To be specific, based on the context–attitude–behavior perspective (29), we delved into the sequential mediating effect of employee job stress and organizational identification in the job insecurity-CWB link. Furthermore, we examined whether CSR activity functions as a buffering factor in the association between job insecurity and job stress.

In line with previous hypotheses and empirical studies, our results showed that employee job insecurity plays a role as a job stressor that substantially increases job stress (18). We found that employee job insecurity negatively influences work behavior. The degree of employee organizational identification is negatively related to his or her CWB (23–25). The overall mediating structure in the link was statistically validated. In addition, we identified CSR activities as a buffering factor explaining the job insecurity-job stress link. In summary, we believe that this research can contribute to the job insecurity literature by bolstering existing studies that showed the detrimental impacts of job insecurity.

Second, we demonstrated that an organization's good and benevolent behavior to benefit society (i.e., CSR activity) functions as a critical contingent variable that moderates the job insecurity-job stress link. From the perspective of an employee, when the organization to which he or she belongs is perceived as reputable and respectable by proactively fulfilling responsibilities for its society, the employee is likely to feel a sense of escalated social self, enhanced pride, and positive perceptions toward the organization. These positive psychological states then directly/indirectly reduce the negative influences of job insecurity. In other words, the positive perceptions or emotions that originate in CSR offset the negative impacts of an unstable job. These results show the importance of organizational-level benevolent activities, which can be measured as level of CSR activities, as well as the necessity of good deeds in dealing with the negative impacts of job insecurity in an organization.

Third, we investigated the influence of job insecurity on employee CWB, as one of the critical 'negative behaviors' in an organization. Although many scholars have described the impact of job insecurity in organizations, previous studies have tended to focus on employees' positive perceptions, attitudes, and behaviors (e.g., organizational identification, employee engagement, job satisfaction, voice/safety behavior, organizational citizenship behavior, and innovative behavior), paying relatively less attention to negative behavior, such as CWB. Given that organizational life includes both positive and negative perspectives, but also that employees' positive and negative behaviors originate in different psychological mechanisms, our attempt to examine the impact of job insecurity on CWB contributes to the job insecurity literature (9, 13).

### Practical implications

This research may provide some practical contributions for top management teams who want not only to understand the impacts of job insecurity on employee behaviors, but also to decrease the negative impacts. First, based on the empirical results of our research, we expect that top management teams could better understand the seriously harmful effects of job insecurity on employee behaviors. We empirically showed that job insecurity substantially increases negative behavior (i.e., CWB), which is closely related to organizational outcomes. Considering that an employee's behaviors tend to be directly associated with organizational performance, the degree of employee job insecurity could critically deteriorate an organization's competitive advantage and sustainability. The current study suggests that top management teams should understand and carefully resolve these important issues based on a variety of rules, incentives, practices, and systems.

Second, the current study also provides direction for top management teams to diminish the negative influence of job insecurity in an organization. We suggest that leaders understand and adequately use the buffering effect of CSR to decrease the harmful results of job insecurity. Top management teams should not only actively implement CSR activities, but also effectively inform the employees of the organization's benevolent actions to aid society. Top management teams should consider the CSR activities as an effective investment instead of a reluctant moral duty. The good and benevolent behaviors of an organization (i.e., CSR) may significantly reduce the negative impacts of job insecurity.

Third, we provide useful indicators or criteria for top management teams who want to monitor and assess the harmful impacts of job insecurity as well as the effectiveness of various buffering factors (e.g., a variety of human resource management systems and practices for reducing the harmful effects of job insecurity). The results of this study demonstrate that degree job stress and organizational identification function as sequential mediators in the job insecurity-counterproductive work behavior link. This means that job stress and organizational identification are important criteria to understand and evaluate how job insecurity influences negative employee behavior. In addition, as aforementioned, the buffering effects of CSR activities can be measured or estimated by assessing change in job stress and organizational identification. In other words, when job stress and organizational identification are not changed after actively implementing CSR activities, this indicates that positive impacts of CSR may not be realized. In sum, we suggest that top management teams monitor the levels of sequential mediators to assess the impacts of both job insecurity and its buffering factors in an organization.

### Limitations and suggestions for future research

Although we believe our research meaningfully contributes to the job insecurity and CWB literature, there are some limitations. First, we could not measure the degrees of job insecurity and CSR activities in an objective manner because we utilized only self-reported survey data that is likely to be subjective. Although we acknowledge that objective phenomena such as amount of CSR investment and downsizing rate are not likely to directly affect employee perceptions, attitudes, and behaviors, the objective measures are likely to be unconsciously reflected in his or her reactions. Therefore, we suggest that future research utilize both subjective and objective measures and compare the differential influences. Second, we did not adequately consider external factors that significantly influence the degree of job insecurity. A variety of objective variables surrounds an employee's perception of his or her job insecurity including downsizing rates, quality or features of HRM systems, and characteristics of the social security system at the national level (7). Thus, we suggest that further research should control for such objective variables.

Third, although the fundamental values that CSR pursues should be universal in Western and Eastern contexts (48, 49), cultural differences are likely to exist affecting employee perceptions toward CSR activities. As South Korea has experienced rapid economic growth, there is a possibility that employees of South Korean firms may react differently to moral activities compared to employees of Western organizations (48). Therefore, the results of the current research should be carefully interpreted.

## Conclusion

Relying on a context–attitude–behavior perspective, we assessed the influence of job insecurity on CWB. Our results demonstrated that job insecurity increases employee CWB *via* the sequential mediating roles of job stress and organizational identification. CSR functions as a positive moderator in the job insecurity–job stress link. These results indicate that the degrees of employee job stress and organizational identification are intermediating processes translating job insecurity into negative behavior. Moreover, CSR activities diminish the negative impact of job insecurity in an organization. Although this research has limitations, the current study can positively contribute to the job insecurity literature.

## Data availability statement

The raw data supporting the conclusions of this article will be made available by the authors, without undue reservation.

## Ethics statement

The studies involving human/animal participants were reviewed and approved by Macromill Embrain Group of Ethics Committee.

## Author contributions

B-JK contributed by writing the original draft of the manuscript and in the conceptualization, data collection, formal analysis, and methodology. JJ, JL, and M-JK contributed in the conceptualization, analysis, revision, and editing the manuscript. All authors have read and agreed to the published version of the manuscript.
